# BREAST CANCER: the educational level of patients correlated with the level of procrastination

**DOI:** 10.1192/j.eurpsy.2022.1696

**Published:** 2022-09-01

**Authors:** C.D. Tabugan, A.-C. Bredicean, C. Giurgi-Oncu, O. Cristina, P. Zsolt, L. Hogea

**Affiliations:** 1 ” Victor Babes” University of Medicine and Pharmacy, Neurosciences, TIMISOARA, Romania; 2 University of Medicine and Pharmacy “Victor Babes” Timisoara, 5. neuroscience Department, Timisoara, Romania; 3 Victor Babes University of Medicine and Pharmacy, Neuroscience, Timisoara, Romania; 4 ONCOHELP, Oncologie, Dumbravita, Romania; 5 ARAD COUNTY EMERGENCY CLINICAL HOSPITAL, Neurosciences, Arad, Romania; 6 “Victor Babes” University of Medicine and Pharmacy, Department Of Neurosciences, Timisoara, Romania

**Keywords:** PROCRASTINATION EDUCATION BREAST CANCER

## Abstract

**Introduction:**

Even if breast cancer is a severe pathology that can cause the death of a person, nowadays there are effective screening methods that could help us to discover in due time the tumor formation and thus be able to benefit from conservative breast surgery.

**Objectives:**

Evaluating the feasible relationship between the noted levels of procrastination and the educational level of subjects

**Methods:**

The analyzed group comprises a number of 152 female subjects (n=152). They were divided in three subgroups: subgroup I(26) composed of women with lower education, subgroup II(66), women with medium education level and subgroup III(60), women with higher education. A socio-demographic questionnaire and the Tuckman Procrastination Scale have been applied.

**Results:**

Comparing the three subgroups, the levels of procrastination were similar. Low levels of procrastination were most common in all three subgroups: in the subgroup I 57,69%, in the subgroup II 56,06% and in the subgroup III 53,33%. Average procrastination levels were observed in 34,61% of women in subgroup I, 42,42% of women in subgroup II and 45% of women in subgroup III. Concerning high levels of procrastination we can affirm that they involve a small number of subjects. Measuring the degree of connection between the two variables, we obtained as a result r=0.13, which means a very weak, non-existent correlation.

**Conclusions:**

The study revealed that there is no relationship between the level of education and the levels of procrastination that include postponing the presentation to the doctor.

**Disclosure:**

No significant relationships.

